# Laser-Induced Crystallization of Copper Oxide Thin Films: A Comparison between Gaussian and Chevron Beam Profiles

**DOI:** 10.1021/acsami.2c11412

**Published:** 2022-10-27

**Authors:** William Bodeau, Kaisei Otoge, Wenchang Yeh, Nobuhiko P. Kobayashi

**Affiliations:** † Electrical and Computer Engineering Department, Baskin School of Engineering, University of California Santa Cruz, Santa Cruz, California 95064, United States; ‡ Graduate School of Natural Science and Technology, Shimane University, Matsue, Shimane 690-8504, Japan; § Nanostructured Energy Conversion Technology and Research (NECTAR), University of California Santa Cruz, Santa Cruz, California 95064, United States

**Keywords:** copper oxide, laser, crystallization, Gaussian, chevron, beam profile, cellular automaton

## Abstract

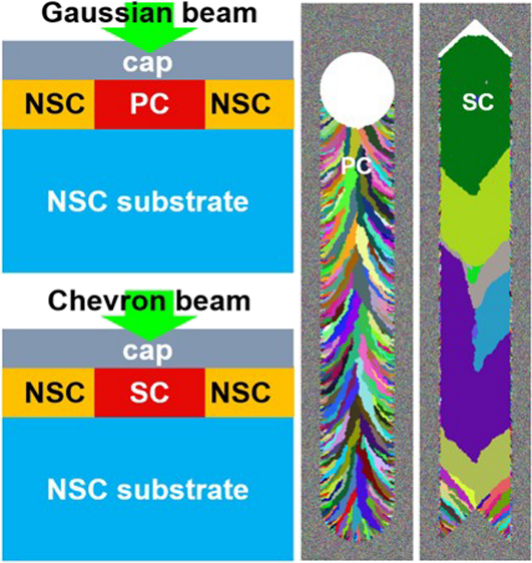

The use of a laser with a Gaussian-beam profile is frequently adopted in attempts of crystallizing nonsingle-crystal thin films; however, it merely results in the formation of polycrystal thin films. In this paper, selective area crystallization of nonsingle-crystal copper(II) oxide (CuO) is described. The crystallization is induced by laser, laser-induced crystallization, with a beam profile in the shape of a chevron. The crystallization is verified by the exhibition of a transition from a nonsingle-crystal phase consisting of small (∼100 nm × 100 nm) grains of CuO to a single-crystal phase of copper(I) oxide (Cu_2_O). The transition is identified by electron back scattering diffraction and Raman spectroscopy, which clearly suggests that a single-crystal domain of Cu_2_O with a size as large as 5 μm × 1 mm develops. The transition may embrace several distinctive scenarios such as a large number of crystallites that densely form grow independently and merge, and simultaneously, solid-state growth that takes place as the merging proceeds reduce the number of grain boundaries and/or a small number of selected crystallites that sparsely form grow laterally, naturally limiting the number of grain boundaries. The volume fraction of the single-crystal domain prepared under the optimized conditions—the ratio of the volume of the single-crystal domain to that of the total volume within which energy carried by the laser is deposited—is estimated to be 32%. Provided these experimental findings, a theoretical assessment based on a cellular automaton model, with the behaviors of localized recrystallization and stochastic nucleation, is developed. The theoretical assessment can qualitatively describe the laser beam geometry-dependence of vital observable features (e.g., size and gross geometry of grains) in the laser-induced crystallization. The theoretical assessment predicts that differences in resulting crystallinity, either single-crystal or polycrystal, primarily depend on a geometrical profile with which melting of nonsingle-crystal regions takes place along the laser scan direction; concave-trailing profiles yield larger grains, which lead to a single crystal, while convex-trailing profiles result in smaller grains, which lead to a polycrystal, casting light on the fundamental question *Why does a chevron-beam profile succeed in producing a single crystal while a Gaussian-beam profile fails?*

## 1 Introduction

Laser-induced-crystallization (LIC) frequently adopted in attempts of crystallizing nonsingle-crystal (NSC) thin films offers attractive features advantageous for functional devices that need to be built on NSC substrates such as glasses for which epitaxial growth, a conventional technique to obtain single-crystal semiconductor thin films, does not serve. LIC has a long history, dating back to the late 1970s^1−5^ with a significant emphasis on elementary semiconductor thin films containing a single chemical element such as Si to develop thin film transistors. LIC was also exploited, to a smaller extent, for semiconductor thin films containing multiple chemical elements (e.g., group IV compound semiconductors, group III-V compound semiconductors, metal oxide semiconductors^6−12^). While conventional LIC frequently adopts a laser with a Gaussian beam profile, uniquely shaped beam profiles were sought to increase the size of crystal grains. For instance, almost 40 years ago, Stultz and Gibbons used a gas-laser with beam profiles shaped into rings and crescents to treat amorphous Si films and showed that the size of crystal grains increased substantially.^13^ Kawamura et al.^200^ used a gas-laser with a beam profile sculpted into a ring and obtained Si grains as large as 50 μm × 600 μm. More recently, Im et al. used a solid-state-laser with a beam profile shaped into a line segment (750 μm long) to prepare poly-Si films made of grains as large as 5 μm × 5 μm.^14^ Kuroki et al. prepared poly-Si films consisting of elongated grains with a length of 100 μm and a width of 2 μm with a laser beam shaped into two 500 μm-long line segments separated by 20 μm.^15^ Furthermore, Nguyen et al. demonstrated poly-Si films by using a laser beam profile containing multiple line segments effectively covering an area of 1.1 μm × 100 μm, producing Si grains with an average size of 20 μm × 2 μm.^16^ However, all these cases merely produced thin films made of multiple crystalline domains (i.e., polycrystal).

In this paper, selective area crystallization of NSC copper(II) oxide (CuO) is described. The crystallization is achieved by LIC with a beam profile in the shape of a chevron, a marked contrast to a Gaussian-beam profile.^17^ The crystallization is verified by observing a transition from an NSC phase consisting of small (∼100 nm × 100 nm) grains of CuO to an SC phase of copper(I) oxide (Cu_2_O). The transition is identified by electron backscattering diffraction (EBSD) and Raman spectroscopy, clearly suggesting that a single-crystal domain of Cu_2_O with size as large as 5 μm × 1 mm develops. Provided this experimental demonstration, a theoretical assessment based on a cellular automaton model, with the behaviors of localized recrystallization and stochastic nucleation, is developed. The theoretical assessment qualitatively predicts the dependence of vital observable features (e.g., size and gross geometry of domains) obtained in the experiment on the key LIC conditions. The theoretical assessment further predicts that differences in resulting crystallinity, either SC or polycrystal, primarily depend on the geometric details with which nonsingle-crystal regions exposed to laser melt in relation to the scan direction of the laser. Concave-trailing profiles yield larger grains, which lead to single-crystal, while convex-trailing profiles result in smaller grains, which lead to polycrystal, casting light on the fundamental question *Why does a chevron-beam profile succeed in producing single-crystal, while a Gaussian-beam profile fails?*

## 2 Experimental Section

### 2.1 Overview of the Experimental Process

**Figure 1:**
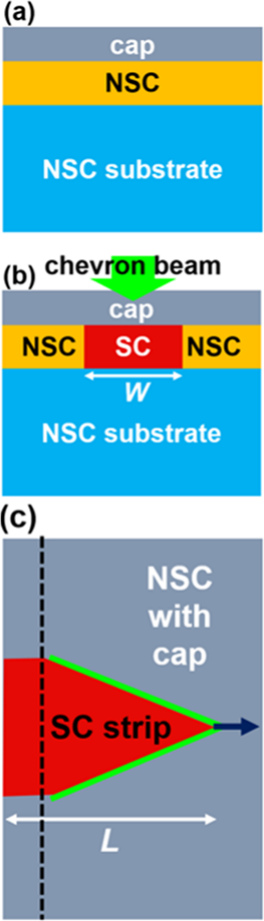
Concept of chevron-beam LIC. (a) Initial structure. (b) Laser with a chevron-beam profile locally melts the nonsingle-crystal (NSC) layer. Upon cooling, the melted region becomes a single crystal (SC) shown as a cross-sectional region in red marked “SC”, (c) a top-view of the SC strip in red in (b) (the cap layer on the strip is partially removed to reveal the SC strip). The laser with a chevron-beam profile is depicted by two green lines joined at their ends. The black arrow represents the direction along which the laser is scanned. The dotted line shows a section along which the cross-sectional region in (b) is drawn. *W* and *L* are the width and length of the SC strip, respectively.

The concept of LIC with a chevron-beam profile, chevron-beam LIC, is depicted in Figure 1. Figure 1a illustrates the initial structure that consists of a nonsingle-crystal layer deposited on a nonsingle-crystal substrate. The nonsingle-crystal layer is covered with a cap layer. In our implementation, the cap layer, the nonsingle-crystal layer, and the nonsingle-crystal substrate were a 200 nm-thick silicon dioxide (SiO_2_) layer, a 130 nm-thick CuO layer, and a fused-silica substrate, respectively. The CuO layer and the SiO_2_ cap layer were deposited sequentially in a single vacuum chamber without breaking the vacuum by DC magnetron sputtering and pulsed DC magnetron sputtering, respectively, at room temperature. A specific thickness of 130 nm was chosen for the CuO layer to ensure sufficient absorption of the laser with the wavelength of 405 nm. The presence of the SiO_2_ cap layer was found to be critical in reducing incongruent evaporation from the underlying CuO layer during chevron-beam LIC. As schematically depicted in Figure 1b, a laser with a chevron-beam profile travels through the SiO_2_ cap layer and locally melts the CuO layer; subsequently, the melted region becomes a single crystal upon solidification as illustrated by the cross-sectional region in red marked “SC”. Figure 1c displays a top view of the single-crystal strip in red (the cap layer on the single-crystal strip is not shown to reveal the single-crystal strip for the display purpose). The laser with a chevron-beam-profile is depicted by two green lines joined at their ends. The black rightward arrow represents the direction along which the laser is scanned. The cross-sectional region of the single-crystal strip in Figure 1b is drawn along the black dotted line in Figure 1c. *W* ≈ 10 μm in Figure 1b and *L* in Figure 1c are the width and length of the resulting single-crystal strip. *W* is comparable to the opening of the chevron-beam profile in green in Figure 1c, while *L* is only limited by the linear translational motion of the moving stage (i.e., the distance over which the laser is scanned) and can be extended as needed.

### 2.2 Deposition and Characteristics of Nonsingle-Crystal CuO Layers

Reactive DC magnetron sputtering was used to prepare nonsingle-crystal CuO layers. The sputtering target of 3 inches in diameter was Cu with a purity of 99.9999%. A mixture of Ar and O_2_ was used as a reactive gas. The flow rate of the reactive gas was fixed to 10 sccm. DC plasma power applied to the Cu target was fixed at 50 W. The substrate temperature was kept at room temperature during the deposition. Figure S1 shows the cathode voltage *V*_c_ plotted as a function of the ratio of flow rate of Ar to that of O_2_ (the Ar/O_2_ flow rate ratio *R*_Ar–O_). Three distinctive regions are seen depending on *R*_Ar–O_. At *R*_Ar–O_ = 1, *V*_c_ was ∼360 V and resulting films were thus copper. As *R*_Ar–O_ was reduced to 0.7, *V*_c_ increased from 360 to 442 V, indicating that the surface of the Cu target was reacted with oxygen, and as a result, the secondary electron emission coefficient decreased and *V*_c_ increased. Under these conditions, however, the resulting copper oxide films did not fully develop stoichiometric CuO as the content of oxygen in the sputtering environment was insufficient, resulting in Cu-rich films (CuO_*x*_, *x* < 1). As shown in Figure S2, since no Raman peaks associated with either single-crystal CuO or single-crystal Cu_2_O was seen in as-deposited CuO_*x*_ layers, the as-deposited CuO_*x*_ layers were found to be amorphous.

### 2.3 LIC with a Chevron-Beam Profile

The chevron-beam profile was established by having the output of a 405 nm wavelength multimode CW laser-diode pass through a one-sided dove prism that converted the line-beam profile into a chevron-beam profile^18^ focused on the CuO layer. The initial structure in Figure 1a was mounted on a linearly moving stage that advanced at a speed (i.e., scan rate *R*_scan_) in the range of 0.4–5 mm/s with respect to the fixed position of the laser with the laser power output *P*_L_ varied in the range of 50–140 mW and thus provided the geometrical dimension of the chevron-beam profile; the areal laser power density was varied in the range of 1–1.5 × 10^6^ W/cm^2^. For each unique LIC condition established by specific *P*_L_ and *R*_scan_, multiple samples, typically 10, were prepared in separate runs to ensure that LIC processes were reproducible within the errors that did not skew statistics of data collected to evaluate the nature of crystallization.

### 2.4 Electron Backscattering Diffraction and Raman Spectroscopy

Electron backscattering diffraction (EBSD) was performed after removing the SiO_2_ cap layer to identify the phase transition from nonsingle-crystal CuO to single-crystal Cu_2_O. EBSD was carried out by using the EBSD detection system TSL-OIM (TSL Solutions Co.) equipped on an SEM system JSM-IT800 (Jeol Ltd.). The acceleration voltage used in the analysis was 15 kV, and the working distance was 15 mm. Raman spectroscopy was conducted with the Reinshaw System 1000s (Renishaw Co.) with the excitation wavelength of 514.5 nm (Ar laser) with a backscattering configuration at room temperature to evaluate crystallinity of the resulting single-crystal strips. The objective lens had a numerical aperture of 0.8.

## 3 Experimental Results and Discussion

**Figure 2:**
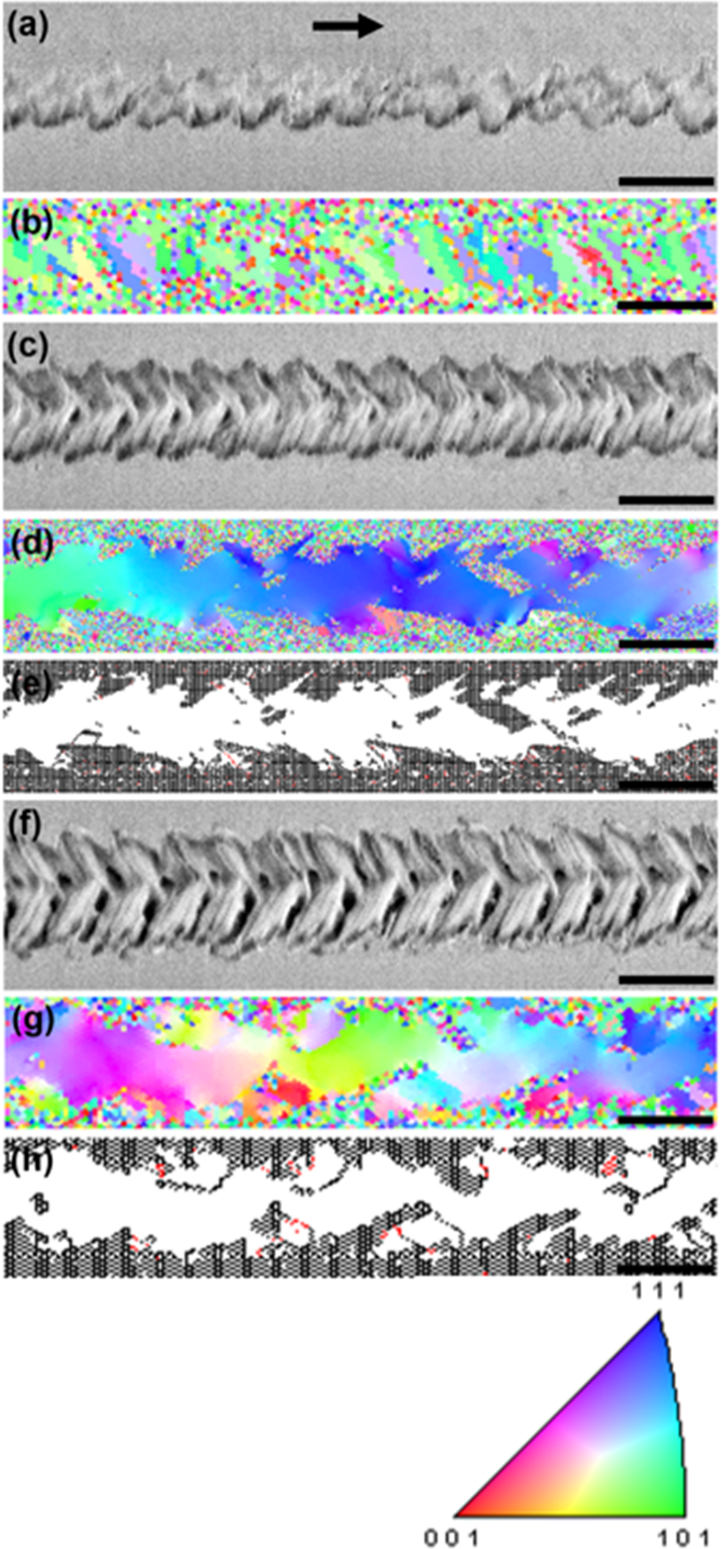
Panels (a, c, f) show images of three strips collected by SEM. The three strips were prepared by the chevron-beam LIC with three different *P*_L_ values: (a) 58.1, (c) 61.2, and (f) 64.3 mW (*R*_scan_ was fixed at 0.4 mm/s). The black arrow in (a) represents the scanning direction of the laser for all the three strips (i.e., for all the panels in this figure). Panels (b, d, g) show crystal orientation maps of the strips prepared at *P*_L_ = 58.1, 61.2, and 64.3 mW, respectively, corresponding to panels (a, c, f). The triangular color map is in reference to the major crystal orientations. For panels (d, g), correlative grain boundary maps are presented in panels (e, h). Discontinuous random angle grain boundaries and coincident site lattice boundaries are shown in black and red. The scale bars represent 5 μm.

Figure 2a,c,f shows images of three strips collected by scanning electron microscopy (SEM) by titling the strips by 70°. The three strips were prepared by the chevron-beam LIC with three different *P*_L_ values: panels (a) 58.1, (c) 61.2, and (f) 64.3 mW (*R*_scan_ was fixed at 0.4 mm/s), respectively. The black arrow in Figure 2a represents the scanning direction of the laser for all the three strips (i.e., for all the panels in Figure 2). Correspondingly, crystal orientation maps, obtained by EBSD to reveal a specific crystal orientation parallel to the surface normal of the three strips, are displayed in Figure 2b,d,g for the strips prepared at *P*_L_ = 58.1, 61.2, and 64.3 mW, respectively. ON the bottem, a triangular color map is provided as a reference showing the major crystal orientations. For the strips prepared at *P*_L_ = 61.2 and 64.3 mW shown in Figure 2d,g, respectively, correlative grain boundary maps, also collected by EBSD, are presented in Figure 2e,h. A grain boundary map is not provided for the strip prepared at *P*_L_ = 58.1 mW in Figure 2b because this strip is found to be a polycrystal for which the presence of grain boundaries is apparent even in its crystal orientation map in Figure 2b. A series of the SEM images in Figure 2a,c,f indicates that distinctive surface features associated with the formation of strips by the chevron-beam LIC amplify in the direction parallel to the scan direction of the laser as *P*_L_ increases. Moreover, the surface features appear more pronounced as *P*_L_ is increased, suggesting that the volume within which the laser and the CuO layer interact, both optically and thermally, increases as *P*_L_ is increased. The crystal orientation map in Figure 2b indicates that the strip prepared at *P*_L_ = 58.1 mW contains numerous grain boundaries running across the strip, resulting in a polycrystal as a whole with the presence of domains with a color that changes discontinuously along the length of the strip. While the strip prepared at *P*_L_ = 58.1 mW in Figure 2a contains randomly oriented grains, the crystal orientation maps of the strips prepared at *P*_L_ = 61.2 and 64.3 mW in Figure 2d,g, respectively, consist of a single domain (i.e., these domains are single-crystal), although the domains seem to rotate along the axes parallel to the length of the strips, which is seen as a continuous change in color along the length. The grain boundary maps in Figure 2e,h further confirm that these strips are single-crystal as no continuous random angle grain boundaries nor coincident site lattice boundaries are found. It is worth mentioning that when *P*_L_ was raised to 67.4 mW, voids appeared within a strip although the strip remained single-crystal.

**Figure 3:**
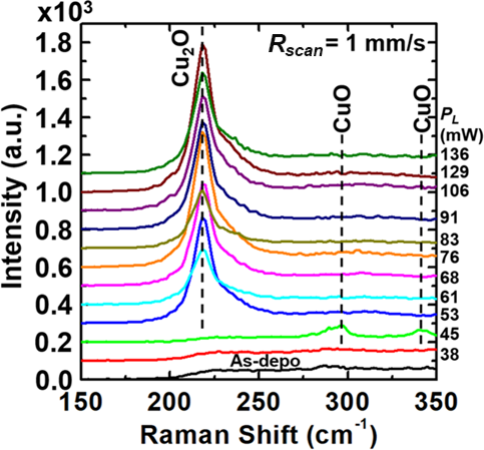
A series of Raman spectra collected from the strips prepared using various *P*_L_ values in the range of 38–138 mW (*R*_scan_ was set to 1 mm/s for all these strips). The spectra of strips prepared at *P*_L_ in the range of 53–138 mW all show the dominant phonon mode at 218 cm^–1^ that represents the second overtone of the phonon mode at 109 cm^–1^ of crystalline Cu_2_O. In contrast, the spectra of the strip prepared at *P*_L_ at 38 and 45 mW evidently lack the Cu_2_O phonon mode at 218 cm^–1^. More specifically, the spectrum of *P*_L_ = 38 mW shows no distinguishable peaks, while the spectrum of *P*_L_ = 45 mW shows two peaks at 296.5 and 341.3 cm^–1^ characteristic to crystalline CuO.

Figure 3 shows a series of Raman spectra collected from the strips prepared using various *P*_L_ values in the range of 38–138 mW (*R*_scan_ was set to 1 mm/s for all these strips). The spectra of strips prepared at *P*_L_ in the range of 53–138 mW all show the dominant phonon mode at 218 cm^–1^ that represents the second overtone of the phonon mode at 109 cm^–1^, an inactive Raman mode that is the only infrared-allowed mode in superb Cu_2_O crystals,^19^ indicating that the strips prepared at *P*_L_ in the range of 53–138 mW bear structural integrity comparable to Cu_2_O formed under conditions near thermal equilibrium.^20^ Characteristic phonon modes associated with CuO (e.g., ∼300 cm^–1^) are not seen in these spectra, confirming that the strips prepared at *P*_L_ in the range of 53–138 mW are predominantly made of crystalline Cu_2_O.^21,22^ The presence of the well-defined second-order overtone at 218 cm^–1^ further indicates that these strips have high crystallographic integrity.^21^ In contrast, the spectra of the strip prepared at *P*_L_ at 38 and 45 mW evidently lack the Cu_2_O phonon mode at 218 cm^–1^. More specifically, the spectrum of *P*_L_ = 38 mW shows no distinguishable peaks, while the spectrum of *P*_L_ = 45 mW shows two peaks at 296.5 and 341.3 cm^–1^ characteristic to crystalline CuO.^23^ For these two peaks, a noticeable tail exists on the side of lower wavenumbers, which indicates that CuO exists in the form of a polycrystal consisting of crystalline grains with a rather wide range of size;^22^ smaller grain sizes result in broader peaks and redshift.^24−26^ Evidently, two phase transitions underwent as *P*_L_ was raised from 38 to 138 mW: the first transition occurring at 45 mW is associated with a transformation of noncrystalline CuO into crystalline CuO, and the second transition taking place at 53 mW is associated with a transition from crystalline CuO to crystalline Cu_2_O. Although complex oxidation kinetics of copper at elevated temperatures often results in the interplay between the two phases, CuO and Cu_2_O, which would contribute to how Raman spectra appear,^27,28^ the observed modes may largely be attributed to Raman selection rules lifted due to point defects such as Cu vacancies commonly present in p-type Cu_2_O.^29^ Nevertheless, the use of higher *P*_L_ promotes the tendency of converting noncrystalline CuO into crystalline Cu_2_O via an intermediate phase of crystalline CuO when *R*_scan_ is appropriately set. Within a strip that is single-crystal, the Raman analysis indicates that the only phase present in the strip is Cu_2_O (i.e., stoichiometric copper(I) oxide); however, the Raman spectroscopy measurement provides data collected from an area (∼1 mm in diameter) much larger than the region where local fluctuation in chemical composition may occur (i.e., stoichiometry is not strictly maintained from one region to another); thus, phases other than Cu_2_O (i.e., Cu_*x*_O, *x* ≠ 2) may exist locally. Although no grain boundaries that cross the width of the strip were observed in Figure 2e,h, the spatial resolution of EBSD may not be good enough to resolve microscopic grain boundaries that would influence physical properties such as electrical and thermal conductivities and contribute to nonradiative-recombination processes; thus, further studies are necessary when applications that depend on these physical properties are envisioned.

**Figure 4:**
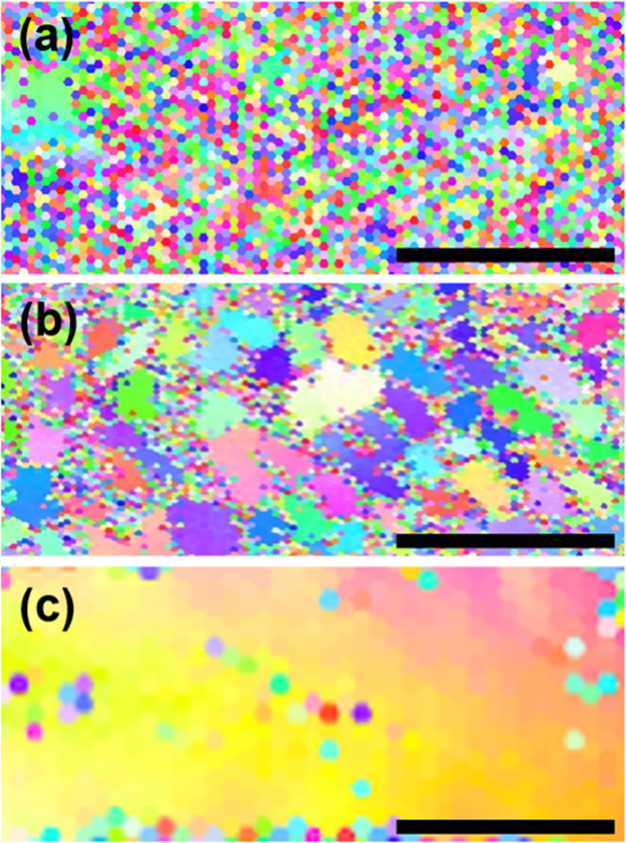
Crystal orientation maps of three strips prepared by the chevron-beam LIC with three different *R*_scan_ values: (a) 10, (b) 5, and (c) 1 mm/s (*P*_L_ was fixed at 87 mW for all these strips). (a) The strip filled with pixels of random colors is deemed noncrystalline. (b) The strip that consists of domains of linear size in the range of 1–2.5 μm is considered to be polycrystal. (c) The strip grew into a single domain filled primarily with yellowish colors—the middle of [001] and [101] on the reference color map provided for Figure 2. The scale bars represent 5 μm.

Shown in Figure 4 are crystal orientation maps of three strips prepared by the chevron-beam LIC with three different *R*_scan_ values: panel (a) 10, panel (b) 5, and panel (c) 1 mm/s (*P*_L_ was fixed at 87 mW for all these strips). Figure 4a is filled with pixels of random colors, indicating that the strip prepared with *R*_scan_ = 10 mm/s exhibits no preferential crystal planes and is deemed noncrystalline. In contrast, the strip in Figure 4b prepared with *R*_scan_ = 5 mm/s consists of domains of linear size in the range of 1–2.5 μm, indicating that individual domains grew laterally although these domains are spatially separated by boundaries that appear to be filled with pixels of random colors (i.e., the presence of grain boundaries); thus, the strip in Figure 4b is regarded as a polycrystal. When *R*_scan_ was decreased to 1 mm/s as in Figure 4c, the strip grew into a single domain (i.e., single-crystal) filled primarily with yellowish colors—the middle of [001] and [101] on the reference color map provided for Figure 2. Figure 4 affirms that, for a given *P*_L_, the use of smaller *R*_scan_ promotes the growth of a single domain, increasing the chance of developing a single-crystal.

**Figure 5:**
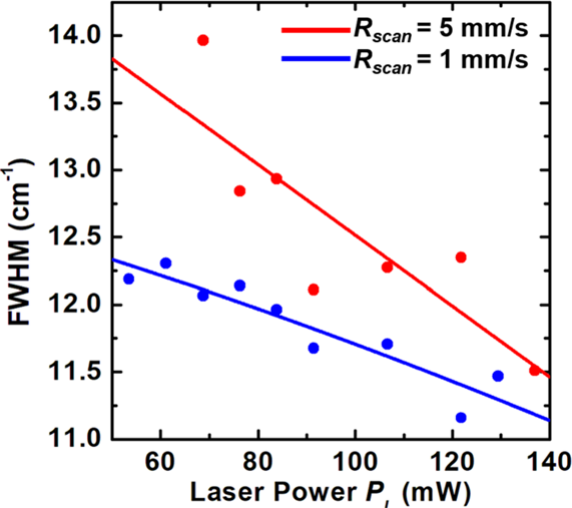
FWHM of the Cu_2_O peak of each spectrum in Figure 3 is plotted as a function of *P*_L_ for two different *R*_scan_ values: 5 and 1 mm/s. For both *R*_scan_ values, the FWHM decreases as *P*_L_ is increased, and the FWHM appears to converge at ∼10.5 cm^–1^ as *P*_L_ exceeds 140 mW regardless of *R*_scan_. This figure clearly suggests that, for a given *R*_scan_, there exists a threshold of *P*_L_ above which the formation of single-crystal Cu_2_O from noncrystalline CuO is energetically preferred.

In Figure 5, the full width at half-maximum (FWHM) of the Cu_2_O peak that appears in each of the Raman spectra shown in Figure 3 is plotted as a function of *P*_L_ for two different *R*_scan_ values: 5 and 1 mm/s. A few features in common for the two *R*_scan_ values reveal that the FWHM decreases, presumably correspoinding to improvements in crystallinity, almost linearly as *P*_L_ is raised, and the FWHM appears to converge at ∼10.5 cm^–1^ as *P*_L_ exceeds 140 mW regardless of *R*_scan_. Figure 5 clearly suggests that, for a given *R*_scan_, there exists a threshold *P*_L_ above which the formation of single-crystal Cu_2_O from noncrystalline CuO is energetically preferred. Once the threshold *P*_L_ is reached (e.g., ∼53 mW for *R*_scan_ = 1 mm/s as seen in Figure 3), crystallinity of Cu_2_O improves (i.e., FWHM decreases), for a given *R*_scan_, as *P*_L_ increases. The interplay between *R*_scan_ and *P*_L_ may be elucidated by considering the ratio *R* of *P*_L_ to *R*_scan_, which has a dimension of J/m (i.e., energy per length). For instance, using an *R*_scan_ of 5 mm/s at 76 mW (i.e., *R* = 15 J/m) results in FWHM of 13.2 cm^–1^, while using an *R*_scan_ of 5 mm/s at 138 mW (i.e., *R* = 27 J/m) results in FWHM of 11.7 cm^–1^, which is comparable to that obtained by using an *R*_scan_ of 1 mm/s at 92 mW (i.e., *R* = 92 J/m). Evidently, the choice of *R*_scan_ for a given *P*_L_ is critical in improving crystallinity, maximizing energy efficiency, and increasing the throughput of the chevron-beam LIC; thus, a theoretical assessment was carried out to illustrate the dependence of the resulting crystallinity on *R*_scan_ and, more importantly, to address the fundamental question *Why does a chevron-beam profile succeed in producing a single crystal while a Gaussian-beam profile fails?*

## 4 Theoretical Results and Discussion

**Figure 6:**
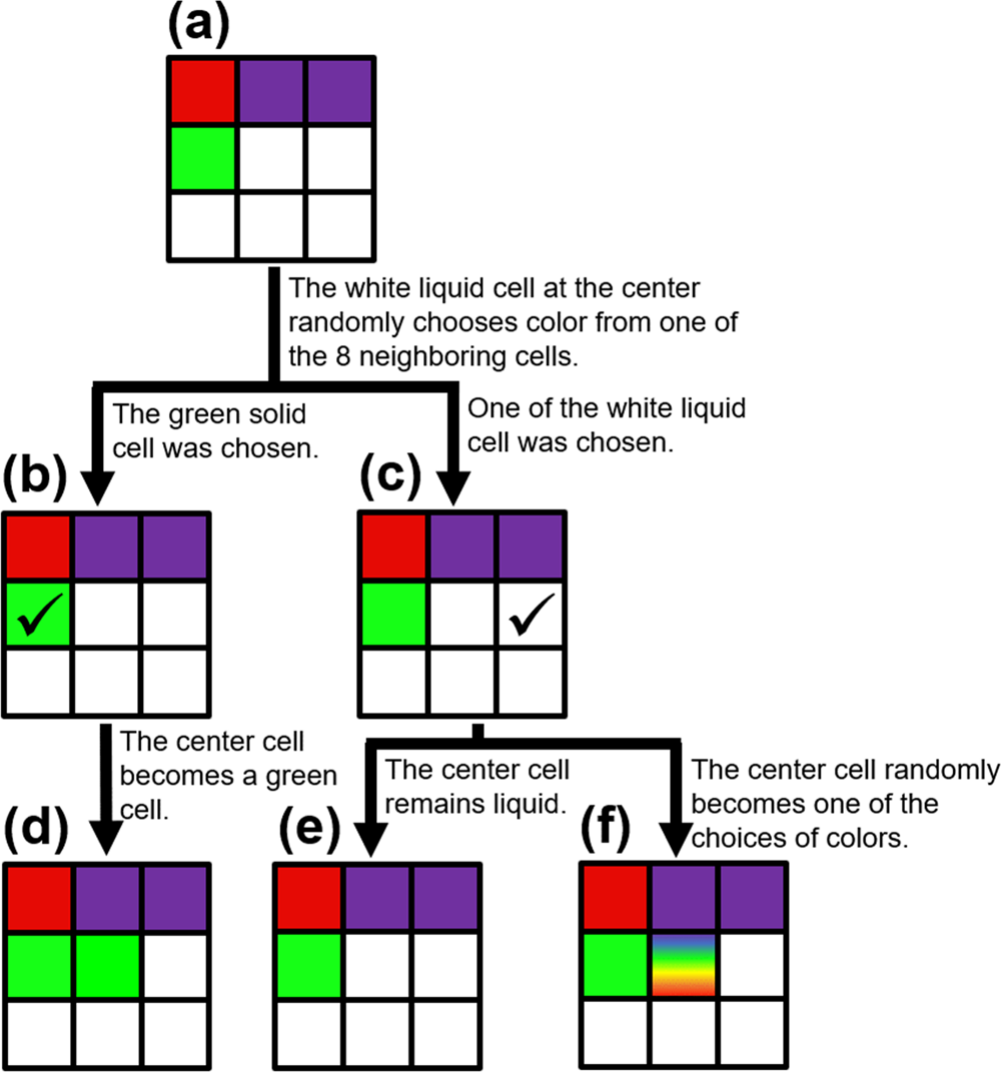
Top view of a nonsingle-crystal thin film that consists of square cells as illustrated in (a). Initially, a random solid state is assigned to each of the cells in the region, producing an effectively nonsingle-crystal initial condition with no distinguishable grains. Within the region, a set of cells illuminated by laser (i.e., cells that are liquid) are shown in white, displaying geometrical details of a laser beam profile. During a time step, a liquid cell (e.g., the center cell in panel (a)) randomly chooses one of the eight surrounding cells and inspects the state of the chosen cell. If the chosen cell is solid as in (b), the updating cell changes its state to match that of the chosen cell as shown in panel (d). If the chosen cell is liquid as in (c), the updating cell either remains liquid as in (e) or changes its state to a random solid state as in (f).

In our efforts of visualizing how the use of a chevron-beam profile and *R*_scan_ contribute to the formation of a single crystal, the crystallization was modeled with a nondifferential cellular automaton (NDCA) evolved on a two-dimensional square grid of cells. Each cell can be configured either in a so-called liquid state (visualized as white) or in one of various solid states, representing variations in crystallographic orientation (visualized as distinct colors). While a solid cell, once formed, remains solid with a distinctive color (unless remelted by laser), all liquid cells have a chance to solidify at every time step. A laser beam, with a specific beam profile, scanned over a sample, as illustrated in Figure 1b,c, is modeled by setting cells located within a high-temperature region defined by the laser to the liquid state, and then the laser is moved at the rate *R*_scan_. In our two-dimensional model, an initial, nonsingle-crystal thin film is defined as a rectangle region, the top view of a nonsingle-crystal thin film, that consists of many square cells as illustrated in Figure 6a (only nine cells are shown in panel (a) for the display purpose.). Initially, a random solid state is assigned to each of the cells in the region. A cell with a specific color represents a solid region with a specific crystallographic orientation; for instance, in Figure 6a, the red cell located at top left corner and the purple cell located at the top right corner represent two regions having two different crystallographic orientations. Thus, a system that consists of cells with colors (except white) randomly assigned to each of the cells at the beginning (i.e., Figure 6a) illustrates a nonsingle-crystal solid (i.e., nonsingle-crystal CuO in referencing the experiment). Within a region made of cells (i.e., the region made of nine cells in Figure 6a), a set of cells illuminated by a laser (i.e., cells that are liquid) are shown in white, specifying geometrical details of a laser beam profile. The initial state in panel (a) has two distinctive paths to take; one path that leads to panel (b) and the other path that leads to panel (c) (a black arrow between two panels represents the direction along which time passes, another way of saying that a chevron-beam profile moves at a given *R*_scan_ during a given time increment). During an increment of time, the liquid cell (e.g., the center cell in panel (a)) randomly chooses one of the eight surrounding cells and inspects the state of the chosen cell. If the chosen cell, the green cell located on the left in the middle row and represented by a check mark in panel (b) in this example, is solid, the liquid cell at the center in panel (b) updates its state to match that of the chosen cell (i.e., the green cell on the left) as shown in panel (d). If the chosen cell (e.g., the cell with a check mark in panel (c)), is liquid, the liquid cell at the center either remains liquid as in panel (e) or changes its state to a random solid state as in panel (f). The rainbow color of the center cell in panel (f) illustrates that, when a liquid cell is chosen (e.g., the white cell with a check mark) as in panel (c), the color of the center cell is randomly chosen from a range of colors that represent a corresponding range of crystallographic orientations.

During a time step, a liquid cell (e.g., the center cell in panel (a)) randomly chooses one of the eight surrounding cells and inspects the state of the chosen cell. If the chosen cell is solid, the updating cell changes its state to match that of the chosen cell as shown in panel (d); this is representative of seeded grain expansion. In contrast, if the chosen cell is liquid as in panel (c), the updating cell either remains liquid as in panel (e) or changes its state to a random solid state as in panel (f), which is governed by the parameter *p*_n_, the probability that local solidification (i.e., nucleation) occurs in a given cell within one time step. High *p*_n_ establishes the condition under which nucleation is preferred, while low *p*_n_ signifies that nucleation is not preferred; thus, a *p*_n_ of 0 has solidification only occurring via interface expansion, and a *p*_n_ of 1 has all liquid immediately solidifying, similar to the amorphous starting conditions. The trajectory of laser scan used in the modeling is linear, which is characterized by laser scan speed *v*_scan_ that has a unit of cell numbers per time step. Because growth is limited to one lattice point per time step, a seeded growth velocity is implicit in the model, on the order of 0.3 cells per time step.

**Figure 7:**
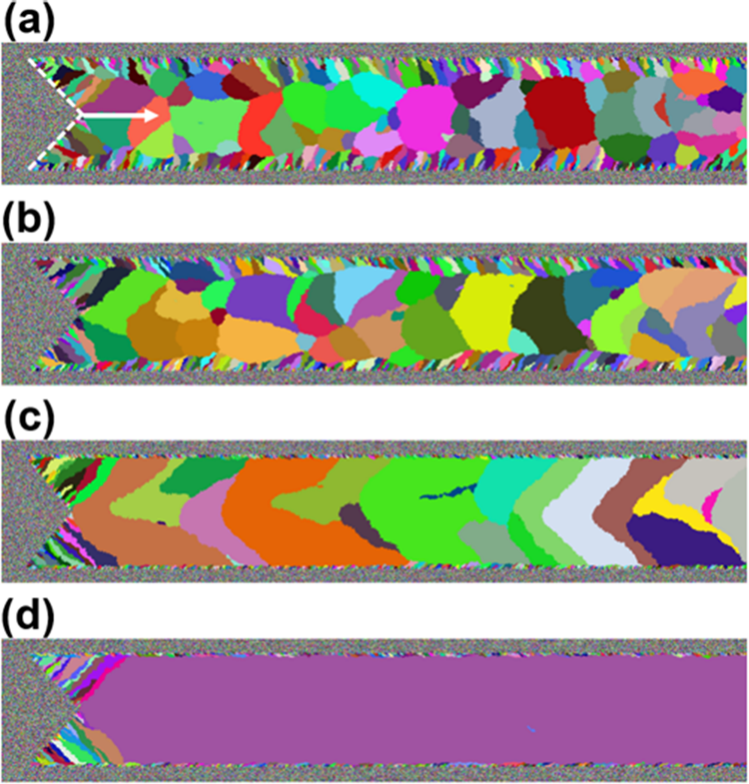
The dependence of the formation of grains on relative *v*_scan_. (a) using *v*_scan_ = 10.0 leads to an apparent decrease in grain size, causing an increasingly thick outer portion made of smaller grains randomly oriented one another. (b) *v*_scan_ = 1.0 and (c) *v*_scan_ = 0.7; using a reduced *v*_scan_ promotes the growth of domains much larger than grains seen (a). (d) using *v*_scan_ = 0.3 (d) results in a single domain (i.e., single crystal). For all cases, *p*_n_ = 5 × 10^–6^.

Figure 7a–d shows the dependence of the formation of grains on relative *v*_scan_. Using a higher *v*_scan_ as in Figure 7a leads to an apparent decrease in grain size, causing an increasingly thick outer portion made of smaller grains randomly oriented with respect to one another. In contrast, as seen in Figure 7b,c, a reduction in *v*_scan_ promotes the growth of domains much larger than grains seen in Figure 7a, and, eventually, a single domain (i.e., single-crystal) forms as *v*_scan_ is further reduced as shown in Figure 7d, which qualitatively suggests that the solid front fails to keep up with the laser being continuously scanned, and a liquid wake begins to form when *v*_scan_ increases, promoting nucleation and decreasing grain size.

**Figure 8:**
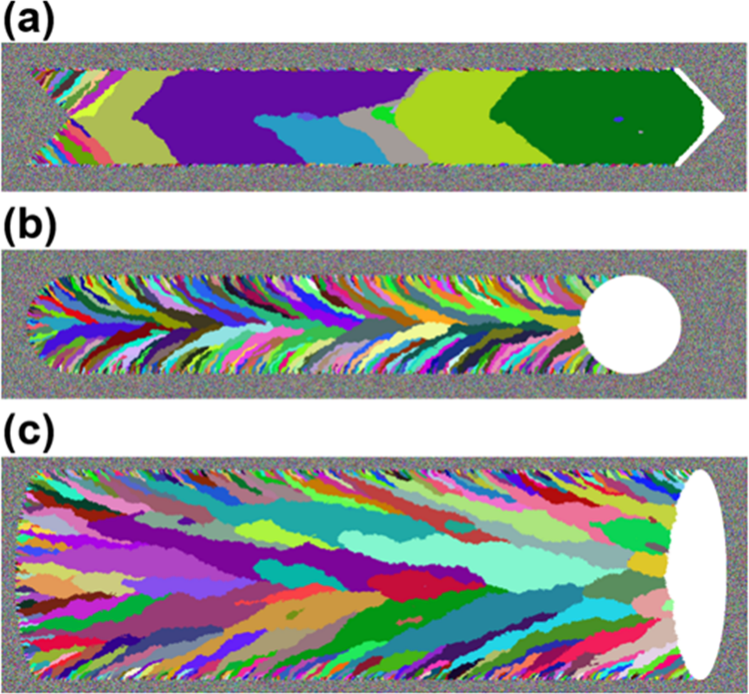
(a, b) Two cases are evaluated for three types of beam profiles: chevron was used in the present study and Gaussian in conventional LIC. The results qualitatively suggest that the Gaussian-beam profile is likely to fail in producing large domains, while the chevron-beam profile offers a better chance of forming a single-crystal, which is consistent with our experiment. (c) An ellipse-beam profile also fails to produce a single domain.

Two cases evaluated for three types of beam profiles, chevron used in the present study and Gaussian in conventional LIC, are compared in Figure 8a,b. As described above, the areas in white illustrate the two types of beam profiles. The results qualitatively suggest that the Gaussian-beam profile is likely to fail in producing large domains, while the chevron-beam profile offers a better chance of forming a single-crystal, which is consistent with our experiment. Detailed analysis of the trailing edge of the Gaussian-beam profile reveals that, as liquid solidifies, grain boundaries progress vertically with respect to the liquid wake, curving forward and in toward the center. In addition, fresh liquid cells were found to be immediately exposed to the unmelted noncrystalline region, causing growth to start as many small randomly oriented grains growing into the channel from the sides, which clearly suggests that any beam profiles presenting a convex tailing liquid wake will result in numerous randomly oriented grains. This was found to be valid to beam profiles generally characterized by a convex curvature as exemplified in Figure 8c that displays an ellipse-beam profile. As far as we are aware, these results explicitly indicate, for the first time, that LIC with a Gaussian-beam profile fails to produce a single-crystal, which is observed in many experimental results of conventional LIC. In contrast, the use of a chevron-beam profile dramatically increases nominal grain size. Detailed analysis reveals that the concave shape of the liquid–solid interface shields the newly created liquid, exposing it only to the recently crystallized region immediately behind it.

**Figure 9:**
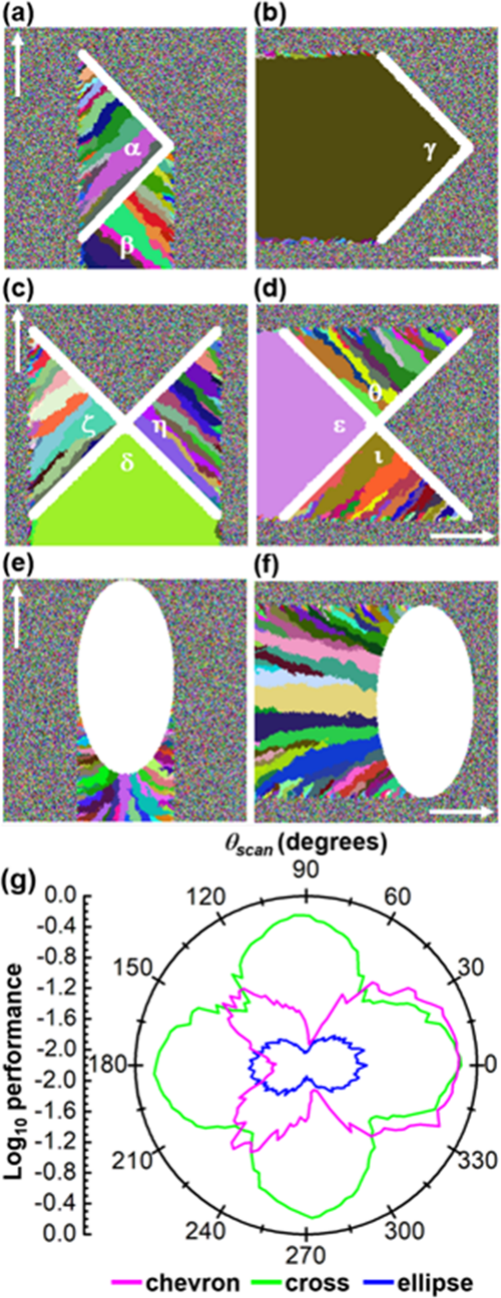
The dependence of solidification on the laser scan direction was examined for various beam profiles. Three types of beam profiles, chevron in (a, b), cross in (c, d), and ellipse in (e, f) were examined. White arrows indicate the direction along which the laser beam was scanned; for instance, in (a), a chevron-beam profile was scanned from the bottom to the top (i.e., scan angle θ_scan_ = 90°), while in (b), a chevron-beam profile was scanted from the left to the right (i.e., θ_scan_ = 0°). (g) Performance defined by the size of polycrystal domains that were averaged over the entire region scanned by a laser and weighted by the total area being scanned by a laser is plotted as a function of θ_scan_ for the three beam profiles. The three plots colored magenta (chevron), green (cross), and dark blue (ellipse) exhibit unique anisotropy resulting from their specific geometrical relationships between a specific beam profile and a scan direction.

The dependence of solidification on the laser scan direction was also quantitatively examined for various beam profiles. Three types of beam profiles, chevron in Figure 9a,b, cross in Figure 9c,d, and ellipse in Figure 9e,f, were examined. In these panels, white arrows indicate the direction along which the laser beam was scanned; for instance, in Figure 9a, a chevron-beam profile was scanned from the bottom to the top (i.e., scan angle θ_scan_ = 90°), while in Figure 9b, a chevron-beam profile was scanted from the left to the right (i.e., θ_scan_ = 0°). Figure 9a,b shows results of using the chevron-beam profile. The two line segments define a chevron-beam profile with a vertex; in other words, two line-segments outline a triangular section with an apex. A polycrystal results both in the triangular section marked with α and in the area denoted with β when the base of the triangular section is parallel to the scan direction as in Figure 9a. Furthermore, the polycrystal sections are made of swaths with diverse colors (i.e., various crystal orientations) that appear to have developed in the direction perpendicular to the line segments. In contrast, a single-crystal forms when the base of the triangular section γ is perpendicular to the scan direction as seen in Figure 9b, indicating the presence of significant anisotropy in terms of resulting crystallinity that substantially depends on the scan direction with respect to the geometry of the beam profile. Figure 9c,d shows results of using the cross-beam profile. Two crossing line segments define four triangular sections with a common vertex located at the cross point of the cross-beam profile. In both Figure 9c,d, a single crystal forms in the triangular section δ and ε with the base perpendicular to the scan direction, which is consistent with Figure 9b. The two triangular sections ζ and η in Figure 9c and θ and ι in Figure 9d with their bases parallel to the scan direction yield a polycrystal made of swaths with diverse colors, which is consistent with the α region in Figure 9a. However, these swaths are eventually converted into a single crystal as the trailing triangular sections δ and ε run over them. Unlike the chevron and the cross-beam profiles, the ellipse-beam profiles in Figure 9e,f fail to produce a single crystal with θ_scan_ = 0 and 90°, which is consistent with Figure 8c. Nevertheless, polycrystal domains that appear in Figure 9e seem to be much smaller than those seen in Figure 9f, clearly illustrating that the size of polycrystal domains strongly depends on the curvature of the ellipse—tighter curvatures result in smaller polycrystal domains.

In Figure 9g, performance defined by the size of polycrystal domains that were averaged over the entire region scanned by a laser and were weighted by the total area being scanned by a laser is plotted as a function of θ_scan_ for the three beam profiles: chevron, cross, and ellipse. The three plots colored magenta (chevron), green (cross), and dark blue (ellipse) exhibit unique anisotropy resulting from their specific geometrical relationships between a specific beam profile and a scan direction. As seen in Figure 9a,b, the chevron-beam profile in magenta exhibits a substantial directional characteristic when the base of the triangular section γ in Figure 9b is perpendicular to the laser scan direction (i.e., θ_scan_ = 0°), while the cross-beam profile in green shows a four-fold rotational symmetry, which arises from the fact that the cross-beam profile is merely a shape made by coupling four chevron-beam profiles with a cross point where four apexes of the four triangles meet. In contrast, the ellipse beam profile in dark blue, with a two-fold rotational symmetry associated with the long and the short axes of the ellipse, seems to be substantially inferior to the chevron-beam profile along all the directions, which is consistent with Figure 9e,f. The collective results shown in Figure 9 suggest that a critical feature required for the formation of single-crystal is a concave beam shape featuring protective side curves trailing out behind the leading central apex.

**Figure 10:**
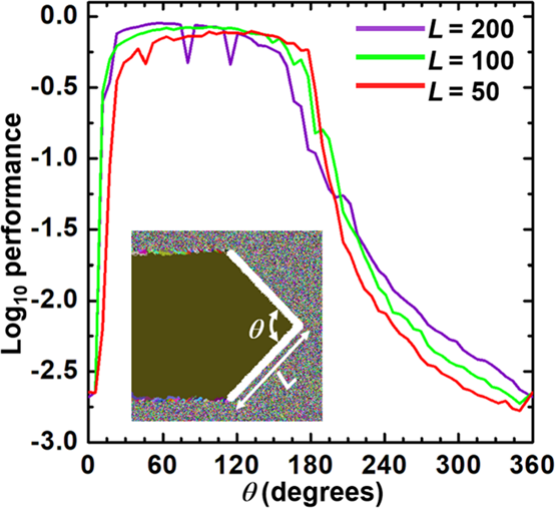
The dependence of the performance on variations in the geometrical factors, length of line segment *L* expressed as the number of cells, and the angle between the two-line segments θ as illustrated in the inset is examined. Chevron-beam profiles with a θ smaller than 180° represent a concave-trailing chevron, while a θ larger than 180° represent a convex-trailing chevron.

Provided advantageous features of the chevron-beam profile, the dependence of the performance on variations in the geometrical factors, length of line segment *L* expressed as the number of cells, and the angle between the two-line segments θ as illustrated in the inset of Figure 10, is examined. In Figure 10, chevron-beam profiles with a θ smaller than 180° represent a concave-trailing chevron, while a θ larger than 180° represent a convex-trailing chevron. The size of the single-crystal domain is independent of *L*, evidently suggesting the importance of the use of a beam profile characterized as concave-trailing to yield a single-crystal.

## 5 Conclusions

The use of a laser with a Gaussian-beam profile is frequently adopted in attempts of crystallizing nonsingle-crystal thin films; however, it merely results in the formation of polycrystal thin films. In this study, selective area crystallization of nonsingle-crystal CuO achieved by LIC with a beam profile in the shape of chevron, chevron-beam LIC with a marked contrast to a Gaussian-beam profile, was demonstrated. The crystallization was verified by observing a transition from a nonsingle-crystal phase of CuO to a single-crystal phase of Cu_2_O with size as large as 5 μm × 1 mm. The use of higher *P*_L_ increases the tendency of converting nonsingle-crystal CuO into single-crystal Cu_2_O via an intermediate phase of crystalline CuO when *R*_scan_ is appropriately set. Nevertheless, a choice of *R*_scan_ for a given *P*_L_ is critical in improving crystallinity, maximizing energy efficiency, and increasing the throughput of the chevron-beam LIC. Provided the experimental demonstration, a theoretical assessment based on a cellular automaton model was developed, which qualitatively predicts the dependence of vital observable features obtained in the experiment. The theoretical assessment further predicts that differences in resulting crystallinity, either single-crystal or polycrystal, primarily depend on the geometric details with which nonsingle-crystal regions are exposed to laser melt in relation to the scan direction of the laser. Concave-trailing profiles yield larger grains, which lead to a single-crystal, while convex-trailing profiles result in smaller grains, which lead to a polycrystal, casting light on the fundamental question *Why does a chevron-beam profile succeed in producing a single-crystal while a Gaussian-beam profile fails?* As far as we are aware, these results explicitly indicate, for the first time, that LIC with a Gaussian-beam profile fails to produce a single-crystal, which is observed in many experimental results of conventional LIC. Given advantageous features of the chevron-beam profile, the dependence of the performance on variations in the geometrical factors was examined, evidently suggesting the importance of the use of a beam profile characterized as concave-trailing to yield a single-crystal.

If successful, our LIC could offer broad societal impacts by offering a new class of single-crystal material platform. For instance, energy conversion and utilization currently dominated by silicon power electronics need such alternative materials as single-crystal wide-band gap semiconductors. In addition, solid-state-lighting, in seeking efficient electrical energy utilization, depends heavily on single-crystal wide-band gap semiconductors. These single-crystal wide-band gap semiconductors are currently prepared by heteroepitaxy, which limits their implementations to applications intended for relatively high cost, significantly limiting its implementation. Our technology, which offers a single-crystal semiconductor without epitaxy, could offer opportunities of vastly expanding the use of single-crystal wide-band gap semiconductors beyond their current implementations. Furthermore, flexible electronics would significantly benefit from our technology. While flexible electronics is mainly adopted for display applications, it continues to expand its applications in medical/healthcare, distribution and retail, and transportation. Current flexible electronics and optoelectronics rely on organic and nonsingle-crystal semiconductors that bear properties inferior to those of single-crystal semiconductors; our technology could offer single-crystal semiconductors for flexible electronics and optoelectronics. The new knowledge described in this paper could lead to benefits of high performance and economies of scalable manufacturing for power electronics, solid-state-lighting, and flexible electronics and optoelectronics, which could drastically change perspectives of these industries.

## Notes

The raw/processed data required to reproduce these findings cannot be shared at this time as the data also forms part of an ongoing study.
